# Deletion of CGLD1 Impairs PSII and Increases Singlet Oxygen Tolerance of Green Alga *Chlamydomonas reinhardtii*

**DOI:** 10.3389/fpls.2017.02154

**Published:** 2017-12-15

**Authors:** Jiale Xing, Peng Liu, Lei Zhao, Fang Huang

**Affiliations:** ^1^Key Laboratory of Photobiology, Institute of Botany, Chinese Academy of Sciences, Beijing, China; ^2^University of Chinese Academy of Sciences, Beijing, China

**Keywords:** *C. reinhardtii*, *x32* mutant, PSII, photo-oxidative stress, singlet oxygen

## Abstract

The green alga *Chlamydomonas reinhardtii* is a key model organism for studying photosynthesis and oxidative stress in unicellular eukaryotes. Using a forward genetics approach, we have identified and characterized a mutant *x32*, which lacks a predicted protein named CGLD1 (Conserved in Green Lineage and Diatom 1) in GreenCut2, under normal and stress conditions. We show that loss of CGLD1 resulted in minimal photoautotrophic growth and PSII activity in the organism. We observed reduced amount of PSII complex and core subunits in the *x32* mutant based on blue-native (BN)/PAGE and immunoblot analysis. Moreover, *x32* exhibited increased sensitivity to high-light stress and altered tolerance to different reactive oxygenic species (ROS) stress treatments, i.e., decreased resistance to H_2_O_2_/or tert-Butyl hydroperoxide (t-BOOH) and increased tolerance to neutral red (NR) and rose bengal (RB) that induce the formation of singlet oxygen, respectively. Further analysis via quantitative real-time PCR (qRT-PCR) indicated that the increased singlet-oxygen tolerance of *x32* was largely correlated with up-regulated gene expression of glutathione-*S*-transferases (GST). The phenotypical and physiological implications revealed from our experiments highlight the important roles of CGLD1 in maintaining structure and function of PSII as well as in protection of Chlamydomonas under photo-oxidative stress conditions.

## Introduction

Microalgae are major source of sustainable biofuel feedstock and CO_2_ sinks for the future (Brennan and Owende, 2010; Scranton et al., 2015). Compared to higher plants, microalgae have advantages in developing such kind of future because these organisms can be cultivated with CO_2_ supply toward reducing greenhouse gas emissions in non-arable areas (Pulz and Gross, 2004; Oey et al., 2013). However, there are limitations in available wild-type strains, including low efficiency of photosynthetic light utilization and high sensitivity to photo-oxidative stress, that impact the algal resource being economically competitive (Wijffels and Barbosa, 2010; Gimpel et al., 2013). To overcome these limitations, generation and characterization of novel mutant strains with desired phenotype is essential.

*Chlamydomonas reinhardtii* (henceforth referred to as Chlamydomonas) is one of the dominant model organisms for studying photosynthesis and oxidative stress in unicellular eukaryotes (Harris et al., 1989; Dent et al., 2015). This is virtually due to its advances in availability of genetic tools for transformation and selection, fully annotated genome (Merchant et al., 2007) that facilitates molecular genetics and ‘omics’ studies toward understanding of fundamental biological processes such as photosynthesis and stress responses (Fischer et al., 2005; Gonzalez-Ballester et al., 2010; Blaby et al., 2013, 2015; Heinnickel and Grossman, 2013; Wu et al., 2015), as well as the processes with biofuel significance including H_2_ photoproduction (Matthew et al., 2009; Chen et al., 2010; Toepel et al., 2013). Also, the organism is haploid capable of heterotrophic growth in the dark and highly sensitive to light, which makes Chlamydomonas extremely ideal for efficient generation and identification of photo-oxidative mutants such as *sor1* and *sak1* (Dent et al., 2001; Fischer et al., 2012; Wakao et al., 2014). The findings based on the studies of the mutants have improved our understanding of cell response in Chlamydomonas to oxidative stress substantially.

Recent advances in phylogenomics have established the GreenCut2 database which reveals 597 nucleus-encoded proteins conserved in plants and algae (Karpowicz et al., 2011). These proteins are presumed to be central to photosynthetic process (Dent et al., 2015). However, functional significance for more than half of these proteins including CGLD (Conserved in the Green Lineage and Diatom) remains to be experimentally determined (Karpowicz et al., 2011). Using forward genetic approach, we have isolated a number of Chlamydomonas mutants with reduced photosynthetic activity, including *msf1* (Zhao et al., 2017) and *x32* which lacks a predicted protein named CGLD1 encoded by Cre02.g084350. Most recent work from *Arabidopsis thaliana* and *Synechocystis* sp. Strain PCC 6803 showed that the homolog proteins of CGLD1 were presumably involved in efficient uptake of Mn^2+^ to thylakoids (Schneider et al., 2016) or homeostasis of Ca^2+^ or Mn^2+^ in Arabidopsis (Wang et al., 2016) or cyanobacterial cells (Brandenburg et al., 2017; Gandini et al., 2017). In contrast, phenotypic and physiological characterization of *cgld1* mutant in Chlamydomonas, which is a key model system for photosynthesis research with great potential in synthetic biology and application (Scharff and Bock, 2014), is still limited (Dent et al., 2015; Schneider et al., 2016). No sufficient information is available regarding functional significance of CGLD1 in Chlamydomonas under various adverse conditions.

Here, we have investigated phenotypic and physiological characteristics of *x32* mutant under normal and photo-oxidative stress conditions. We show that loss of CGLD1 resulted in minimal photoautotrophic growth and PSII activity in Chlamydomonas. We also show that the amount of PSII core proteins and PSII complex was drastically reduced in *x32*. Furthermore, we found that *x32* mutant exhibits increased photoinhibition and altered tolerance to different reactive oxygen species (ROS) treatments. The increased tolerance to singlet oxygen could be largely attributed to up-regulated gene expression of glutathione-*S*-transferases (GST) in the mutant under such conditions.

## Materials and Methods

### Strains, Culture Conditions, and Stress Treatments

*Chlamydomonas reinhardtii* wild-type strains CC400 (mt-), 137c (mt+), CC4051 (mt+) and *cgld1* mutant (strain CAL029_02_05) was obtained from the Chlamydomonas Genetics Center^1^. The *x32* mutant was isolated from an insertion mutant library constructed in our laboratory (Zhao et al., 2017) described in more detail below. The algal cells were cultured in TAP (Gorman and Levine, 1965) or in high-salt minimal (HSM) medium under continuous cool-white fluorescent light (60 μmol photons m^-2^ s^-1^) at 25°C. For all experiments, cells were grown to mid-exponential phase and harvested by centrifugation followed by adjusting the cell density to 2–4 × 10^6^ cells ml^-1^. High-light stress treatment (1,300 μmol photons m^-2^ s^-1^) was performed according to (Zhao et al., 2013) except that the culture was transferred to flasks (25 ml) and the cell density was adjusted to 2 × 10^6^ cells ml^-1^. ROS stress treatments were done as described (Chen et al., 2016). For mRNA and protein analysis, cells were harvested by centrifugation at 2500 *g* (4°C) for 5 min. After washing once with 0.01 M sodium phosphate buffer (pH 7.4), the cell pellets were stored at -70°C.

### Mutant Library Construction and Mutant Isolation with Chlorophyll Fluorescence

The insertion mutant library construction and photosynthetic mutants screen were described (Zhao et al., 2017). Briefly, the wild-type strain (CC400) was used and the mutant library was constructed by transforming this strain via the glass bead method with K*pn*I linearized plasmid pSI103 containing the *aphVIII* gene conferring paromomycin resistance (Kindle, 1990). Transformants that grew on TAP plates with 10 μg ml^-1^ paromomycin (Sigma) were isolated for photosynthetic mutants screening. Mutant screening was based on chlorophyll *a* fluorescence measurements. Sample preparation was done as previously (Zhao et al., 2013) and the measurements were taken with a chlorophyll fluorometer (Maxi-Imaging PAM; Walz, Effeltrich, Germany) by following the manufacturer’s instructions. Among the mutants with both the lowest *F*_v_/*F*_m_ and Y(II) values, *x32* was chosen for subsequent characterization.

### Oxygen Evolution Rate, 77K Fluorescence Emission Spectra and P700 Absorbance Measurements

Oxygen evolution rate of Chlamydomonas was measured as previously described (Sun et al., 2013) with a Chlorolab-2 oxygen electrode (Hansatech, Norfolk, United Kingdom). 77K fluorescence emission spectra were measured with a fluorescence spectrophotometer (F-2500; Hitachi, Japan) as described (Yang et al., 2014) with minor changes (Zhao et al., 2017). An excitation wavelength of 435 nm (5 nm bandwidth) was used to induce chlorophyll *a* fluorescence. The emission spectra were recorded (600–750 nm) and normalized at 716 nm. Light-induced redox changes of P700 were monitored by measuring absorbance at 820 nm using PAM101 fluorometer equipped with a dual-wavelength P700 unit (ED800T). Sample preparation was done according to Berry et al. (2011). A far-red light illumination (FR, 720 nm, 24 μmol photons m^-2^ s^-1^) was provided for 45 s to enable oxidation of P700 to a steady state then turned off to monitor the initial rate of P700^+^ dark reduction according to Klughammer and Schreiber (1998).

### Genetic Analysis and Complementation

Genetic analysis and complementation was done as described (Zhao et al., 2017) with minor modifications. For DNA blot analysis, genomic DNA was isolated from wild-type (CC400) and *x32* mutant using the Plant Genomic DNA Kit by following the manufacturer’s instructions (Tiangen Biotech; Beijing, China). About 10 μg of genomic DNA was digested overnight with the restriction endonucleases *Kpn*I and *Hind* III (New England Biolabs). The fragments resulting from the digestion were separated by 0.8% agarose gel electrophoresis followed by blotting onto nitrocellulose membranes and hybridizing with the DIG high prime DNA labeling and detection starter kit II from Roche (Catalog NO. 11585614910). The detection was achieved using a chemiluminescent substrate CSPD (Disodium 3-(4-methoxyspiro {l,2-dioxetane-3,2′-(5′-chloro)tricyclo[3.3.1.1^3,7^]decan}-4-yl) phenyl phosphate) (Catalog NO. 11 755 633 001).

For gene mapping, genomic DNA flanking the *aphVIII* gene was isolated using high efficiency TAIL-PCR with the specific primers listed in Supplementary Table 1 according to (Liu and Chen, 2007) with slight modifications. The primary amplification reactions (20 μL) was composed of 2 μL of PCR buffer (Takara Bio Inc, Otsu, Shiga; Japan), 200 μM of dNTPs (TransGen Biotech; Beijing, China), 1 μM of any of the LAD primers, 0.3 μM of SP0, 0.5 μL of LA Taq (Takara Bio Inc, Otsu, Shiga; Japan) and 20–30 ng of DNA. Each 25-μL secondary reaction contained 2.5 μL of PCR buffer, 200 μM each of dNTPs, 0.3 μM of AC1 and SP1, 0.5 μl of LA Taq, and 1 μL of 50-fold diluted primary product. The amplified products from the secondary reactions were analyzed by agarose gel electrophoresis and were purified prior to sequencing. Sequencing reactions were performed by Sunbiotech (Beijing Sunbiotech; China) and the data were used to search the Chlamydomonas genome.

For tetrad analysis, genetic crosses between *x32* and the wild-type (137c, mt+) and zygote dissection were performed accordingly to (Harris et al., 1989). The mutant was backcrossed twice and four progeny from distinct zygotes of the second generation (T2-*x32*) were obtained. To complement the *x32* mutant, the gene was amplified from wild-type (CC400) with primers listed in Supplementary Table 1. The amplification product was digested with *Nde*I and *EcoR*I and subcloned into similarly treated vector pDble (Fischer and Rochaix, 2001). The constructed plasmids were introduced into *x32* mutant by transformation. The transformants were then analyzed by chlorophyll fluorescence measurements. Colonies that displayed a wild-type phenotype were also analyzed for the integration of *x32* by PCR with the specific primers listed in Supplementary Table 1.

### Production of Antiserum against Recombinant CGLD1 Protein

Cloning and heterologous expression were performed as previously described (Chen et al., 2010). Total RNA isolation/purification and reverse transcription reactions were done as described (Sun et al., 2013). Briefly, the coding region of the *CGLD1* gene without transmembrane sequence was amplified by PCR with PrimeSTAR HS DNA Polymerase (Takara, Ohtsu, Japan) using specific primers Pet28-CGLD1-F/R (Supplementary Table 1). The amplified fragment was cloned directly into p*Easy*-blunt vector (Beijing TransGen Biotech, China), which was then transformed into competent *Escherichia coli* DH5α cells. Positive clones containing the recombinant plasmid were selected and sequenced to ensure the authenticity of the ORFs (Beijing Sunbiotech, China). Extraction and purification of *E. coli* proteins were done as described (Zhou et al., 2008). A rabbit serum was produced by MBL (MBL, Nagoya, Japan) using the purified recombinant CGLD1 protein as immunogen. Specificity of the antibody was verified by immunoblotting using proteins extracted from the *E. coli* and Chlamydomonas cells, respectively.

### Protein Extraction, BN-PAGE, SDS-PAGE, and Immunoblot Analysis

Cells breakage, isolation of membrane proteins for blue-native (BN)/SDS-PAGE and SDS-PAGE were done as described (Chen et al., 2016). BN gel was prepared and electrophoresis was performed at 4°C with the running program previously reported (Yang et al., 2014) and immunoblot analysis were done according to (Zhao et al., 2013, 2017). The antibodies against D1, D2, CP43, CP47, Cytb_6_f, and AtpB were from Agrisera and those against PsaB, Lhcb4, and LhcII were from J.-D. Rochaix (University of Geneva). The dilutions for the specific antibodies used in this study were: anti-CGLD1 (1:1000), anti-CP43 and CP47 (1:3000), anti-AtpB (1:4000), anti-D1 and D2 (1:5000), anti-PsaB, Cytb_6_, LhcII and Lhcb4 (1:10000). The immuno-signal was detected using the Pro-light HRP ECL detection system (Tiangen Biotech; Beijing, China). The blots were scanned using a UMAX Power-Look 2100XL scanner (Willich, Germany). Protein content was determined according to Peterson (1977) using BSA as standard.

### qRT-PCR Analysis and Enzyme Activity Assay

Quantitative real-time reverse transcription-PCR (qRT-PCR) was done as described (Zhao et al., 2013) using *CBLP* as the internal control. Gene-specific PCR primer pairs for *psbA* and *psbC* were designed using the online program Primer3^2^. The primer pairs previously reported for *APX1, CAT1, GSTS1*, *GSTS2*, and *GPXH* (Fischer et al., 2012; Pokora et al., 2017) were used and listed in Supplementary Table 1. Relative abundance was expressed as the fold change in expression level relative to the reference, calculated as 2^-ΔΔ^*^C^*_T_.

For enzyme activity assay, crude extracts preparation and determination of SOD and CAT activity was done as described (Chen et al., 2016).

## Results

### Phenotypic and Photosynthetic Characteristics of *x32* Mutant

**FIGURE 1:**
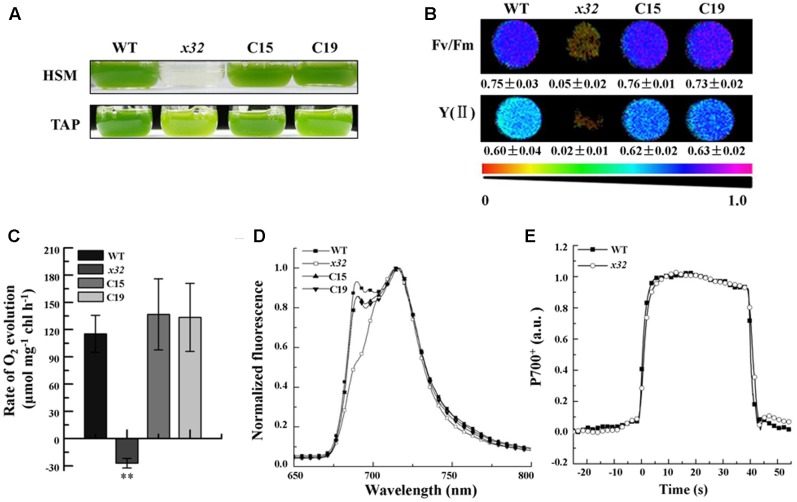
Growth and photosynthetic characters of wild-type (WT), *x32* and the complemented strains (C15 and C19). **(A)** Growth in HSM and TAP medium with an irradiance of 60 μmol photons m^–2^ s^–1^ for 6 and 3 days, respectively. **(B)** Maximal (*F*_v_/*F*_m_) and effective quantum yield of PSII (YII) of the indicated strains. Standard deviations were estimated from three biological replicates. All experiments were repeated twice with similar results. **(C)** Rate of O_2_ evolution in different strains. Standard deviations were estimated from three biological replicates. All experiments were repeated twice with similar results. ^∗∗^*p*-value < 0.01 in Student’s *t*-test. **(D)** 77K fluorescence emission spectra of the indicated strains. The spectra were normalized at 716 nm. All experiments were repeated twice with similar results. **(E)** Light-induced redox kinetics of P700 in wild-type (WT) and *x32* mutant. All experiments were repeated twice with similar results.

The *x32* mutant was isolated during a genetic screen for Chlamydomonas insertion mutants with decreased photosynthetic activity as described previously (for details see Zhao et al., 2017). **Figure 1** shows that, compared to wild-type, growth of *x32* in TAP and high salt minimal (HSM) medium (**Figure 1A**) was severely repressed, suggesting impaired photosynthesis in the mutant. This was confirmed by the measurements of *in vivo* chlorophyll *a* fluorescence (Imaging PAM, Heinz Walz, Germany). The maximal and effective quantum yield of PSII, *F*_v_/*F*_m_ and Y(II), was reduced to the minimal (6.6 and 3.0% of the wild-type level, respectively) (**Figure 1B**). In line with these, the rate of photosynthetic oxygen evolution in *x32* mutant was undetectable (**Figure 1C**). To obtain further functional insights of photosystems in *x32*, low temperature fluorescence (77K) emission spectra of *x32* and wild-type cells were then compared (**Figure 1D**). A differential reduction of the fluorescence emission peak at 686 nm, which is characteristic for PSII mutants (Össenbuhl et al., 2004), was observed in *x32* (**Figure 1D**). This indicates the level of functional PSII in the mutant was significantly lower than wild-type. Since no difference in the light-induced redox kinetics of PSI was revealed between wild-type and the mutant (**Figure 1E**), we conclude that only PSII is affected in the Chlamydomonas mutant *x32*.

### The *x32* Mutant Is Deficient in CGLD1 Protein

**FIGURE 2:**
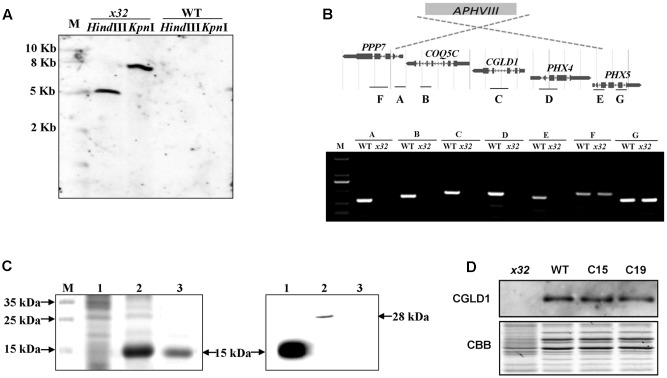
Genetic analysis of the *x32* mutant. **(A)** DNA blot analysis of wild-type and *x32*. Genomic DNA was digested with *Hind*III and *Kpn*I, fractionated by agarose gel electrophoresis and hybridized with a DNA probe of 407bp of *APHVIII* gene. **(B)** Mapping of the deletion in *x32* caused by insertion of the paromomycin resistance cassette (*APHVIII*) by comparative analysis of wild-type and *x32* DNA using PCR specific primer pairs. **(C)** Heterologous expression of recombinant CGLD1 protein and specificity of CGLD1 antibody. Coomassie-stained SDS-PAGE gel separating proteins extracted from *Escherichia coli* cells (left panel). M, protein molecular mass marker. 1 and 2, total proteins without or with isopropyl β-D-1-thiogalactopyranoside (IPTG) induction, respectively. 3, recombinant CGLD1 purified using Ni-NTA column. Specificity detection of the CGLD1 antibody by immunoblotting (right panel). Lane 1, purified recombinant CGLD1 protein (0.02 μg). Lanes 2 and 3, proteins (20 μg) extracted from wild-type and *x32*, respectively. **(D)** Immunoblot detection of CGLD1 in wild-type, *x32* and the complemented strains C15 and C19. Membrane proteins (20 μg per lane) were separated by SDS-PAGE (16%) followed by immunoblotting with the CGLD1 antibody.

DNA blot analysis revealed a single insertion of the paromomycin resistance cassette in the genome of *x32* (**Figure 2A**). The insertion site was mapped on the genome of Chlamydomonas via sequencing the flanking regions of the insert through thermal asymmetric interlaced PCR (Liu and Chen, 2007). Analysis of this genomic region in *x32* revealed a DNA rearrangement that resulted in the deletion of five genes, i.e., Cre02.g084250, Cre02.g084300, Cre02.g084350, Cre02.g084400, and Cre02.g084450 (**Figure 2B**). Prediction by PredAlgo software^3^ shows that only the gene Cre02.g084350 encoding CGLD1 in the GreenCut2 (Karpowicz et al., 2011) is localized in the chloroplast. We therefore introduced the gene into *x32* for complementation. The *x32* mutant phenotype could be fully rescued in the complemented strains (C15, C19) based on growth, *F*_v_/*F*_m_ and Y(II) values (**Figure 1A**). Analysis of genetic crosses between *x32* mutant and wild-type was in agreement with these data (Supplementary Figures 1A,B). The photosynthetic defect of *x32* co-segregated with paromomycin resistance in six complete tetrads analyzed, suggesting that the phenotype is linked to this genetic disruption.

To further confirm the candidate gene, we compared the phenotype of *x32* and another *cgld1* mutant obtained from the Chlamydomonas Resource Center^4^, which was derived from wild-type strain CC4051 with an insertion in exon2 of the *CGLD1* gene (Dent et al., 2015; Schneider et al., 2016), in the presence of additional manganese (Mn^2+^) and calcium (Ca^2+^) (Supplementary Figure 1C). The similar results of growth profiles and *F*_v_/*F*_m_ values of the two *cgld1* mutants (Schneider et al., 2016; Supplementary Figure 1C) verify that disruption of *CGLD1* is the cause of the phenotype observed in *x32.*

In the genome of Chlamydomonas, CGLD1 is annotated as a predicted protein (Phytozome v12.1^5^). To determine the expression of CGLD1 protein in wild-type Chlamydomonas and verify the lack of CGLD1 in *x32*, we generated an antibody against recombinant CGLD1 protein and verified its specificity by immunoblotting using protein extracts from the wild-type Chlamydomonas (**Figure 2C**). Heterologous expression and purification of the recombinant CGLD1 protein was performed as previously reported (Zhou et al., 2008; Sun et al., 2013; Zhao et al., 2017). As shown in **Figure 2C**, the *E. coli* Transetta (DE3) cells produced a substantial amount of the expected recombinant protein possessing an estimated molecular mass of 15 kDa. This corresponds to the molecular mass calculated from the coding region of Chlamydomonas *CGLD1* gene without the transmembrane sequences (**Figure 2C**, left panel). Total proteins extracted from the *E. coli* cells and Chlamydomonas cells were analyzed by immunoblotting with the antibody. A single band at 28 and 15 kDa was detected in wild-type Chlamydomonas and *E. coli* expressing the recombinant CGLD1 protein (**Figure 2C**, right panel), respectively. The former corresponds to the molecular mass that was calculated from the coding sequence of *CGLD1* gene in Chlamydomonas. Since no band was detected in *x32* mutant (**Figure 2C**, right panel), this antibody was therefore used for quantification of CGLD1 in wild-type, *x32* and the complemented (C15, C19) strains (**Figure 2D**).

### Reduced Accumulation of PSII in *x32* Mutant

**FIGURE 3:**
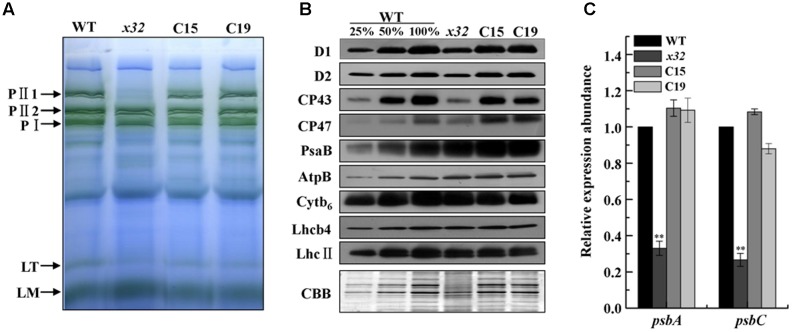
Abundance of photosynthetic proteins and complexes in thylakoid membranes of WT, *x32* and the complemented strains (C15 and C19). **(A)** BN-PAGE of the thylakoid membranes. DM-solubilized membrane proteins (25 μg per lane) were separated by BN-PAGE. PII1, PII2, PSII supercomplexes; PI, PSI-LHCI complex; LT, LHCII trimer; LM, LHCII monomer. Similar results were obtained in two independent experiments. **(B)** Immunoblot detection of various photosynthetic proteins. Membrane proteins (10 μg per lane) were separated by 12% SDS-PAGE followed by immunoblot detection of indicated proteins. A dilution series of wild-type protein extract was used to quantify the amount of proteins in *x32*. Similar results were obtained in at least three independent experiments. **(C)** qRT-PCR analysis of mRNA levels of *psbA* and *psbC* in the indicated strains. Standard deviations were estimated from three biological replicates. Similar results were obtained in three independent experiments. *CBLP* gene was used as a control. ^∗∗^*p*-value < 0.01 in Student’s *t*-test.

The decreased PSII activity of *x32* could be due to reduced accumulation of thylakoid membrane proteins. To clarify this, we analyzed membrane protein complexes of *x32* and wild-type cells by blue-native (BN)/SDS-PAGE using a slightly modified protocol (Yang et al., 2014) as described (Chen et al., 2016). As shown in **Figure 3A**, the overall BN-gel profile of thylakoid membranes isolated from wild-type was similar to earlier reported (Dewez et al., 2009; Chen et al., 2016), demonstrating high technical reproducibility of the BN-PAGE from different experiments. Comparision of the BN-PAGE gel pattern between wild-type and *x32* revealed a clear reduction of the band corresponding to a PSII supercomplex, PII1 containing core subunits of PSII (D1, D2, CP43, CP47) (Rexroth et al., 2003; Dewez et al., 2009), in the mutant (**Figure 3A**). This was further confirmed by immunoblotting using the antibodies specific for key proteins of photosynthetic apparatus (**Figure 3B**). The levels of core subunits of PSII (D1, D2, CP43, and CP47) in *x32* were 30% or less of that in wild-type whereas the levels of the key subunits of PSI (PsaB), Cyt*b*_6_*f* (Cyt*b*_6_), and ATP synthase (AtpB) as well as the LHCII proteins (LhcII and Lhcb4) remained unchanged in *x32* (**Figure 3B**). To test whether the reduced levels of the core PSII proteins in *x32* was due to limited transcription, we then compared the *psbA* and *psbC* mRNA levels between wild-type and *x32* by quantitative real-time PCR (qRT-PCR) analysis. The transcript level of both genes was decreased 67.2% (*psbA*) and 68.3% *(psbC*) in relation to the wild-type (**Figure 3C**). Thus, these experimental data demonstrate a significant and specific impact of CGLD1 on PSII at both mRNA and protein level in Chlamydomonas.

### Increased Sensitivity to High-Light and Peroxide Stress in *x32*

**FIGURE 4:**
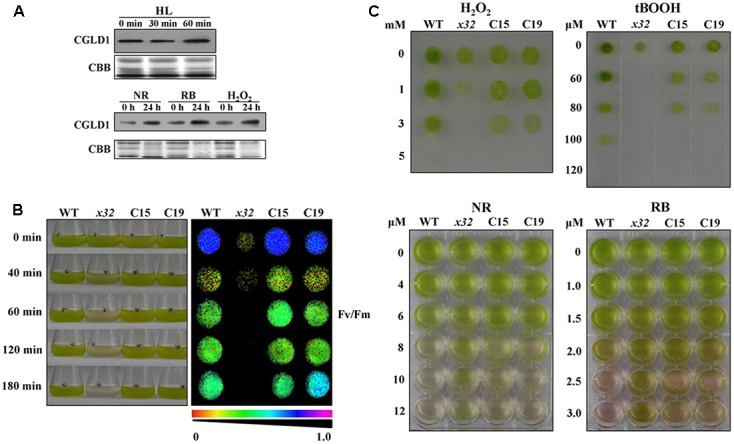
High-light and ROS-stress tolerance of WT, *x32* and the complemented strains (C15 and C19). **(A)** Immunoblot analyses of CGLD1 amounts in Chlamydomonas under high-light (HL; 1,300 μmol photons m^–2^ s^–1^) and ROS-stress conditions. Membrane proteins (10 μg per lane) were separated by SDS-PAGE (16%) followed by immunoblotting with the CGLD1 antibody. Similar results were obtained in three independent experiments. **(B)** Growth and quantum yield of PSII (*F*_v_/*F*_m_) of the indicated strains under high-light treatment (1,300 μmol photons m^–2^ s^–1^). Similar results were obtained in at least three independent experiments. **(C)** ROS tolerance of the indicated strains. Growth recovery after treatment with H_2_O_2_, t-BOOH (tert-Butyl hydroperoxide) followed by spotting on TAP plates for 3 days (upper). Growth after treatment with NR (neutral red) and RB (rose bengal). Similar results were obtained in at least three independent experiments.

To determine potential physiological roles of CGLD1 protein in Chlamydomonas under adverse condition, we performed various stress experiments. Because high-light is the most common stress condition occurring to photosynthetic organisms and Chlamydomonas is a key model system for studying photo-oxidative stress in unicellular eukaryotes, we compared CGLD1 levels of wild-type cells after treatments with high-light (1,300 μmol photons m^-2^ s^-1^) and different ROS, i.e., H_2_O_2_, neutral red (NR) and rose bengal (RB). The stress treatments were done as described (Zhao et al., 2013; Chen et al., 2016) and the CGLD1 protein was monitored using immunoblot analyses. As shown in **Figure 4A**, the amount of CGLD1 was in most cases increased after these stress treatments, indicating that this protein is also involved in photoinhibition/photoprotection against photo-oxidative stress in the organism. To confirm these, we then investigated the sensitivity of *x32* to high-light stress treatment (**Figure 4B**). As expected, cell growth of *x32* was significantly repressed during high-light stress treatment (1,300 μmol photons m^-2^ s^-1^) compared to wild-type (**Figure 4B**). The remarkable reduction of cell growth was observed upon exposure of *x32* to high light for 40 min. This was more apparent based on *F*_v_/*F*_m_ values (**Figure 4B**, right panel), suggesting increased photoinhibition in the mutant.

To determine whether the tolerance of *x32* mutant to oxidative stress was changed due to loss of CGLD1, we also compared the sensitivity of *x32* and wild- type cells to different ROS treatments. Similar results were obtained with wild-type strain in the present (**Figure 4B**) and earlier investigations (Chen et al., 2016). However, the *x32* mutant was more sensitive to H_2_O_2_ and tert-Butyl hydroperoxide (t-BOOH) than wild-type (**Figure 4C**, upper panel). Correlated with this, both gene expression of ascorbate peroxidase 1 (*APX1*) and catalase 1 (*CAT1*) (Supplementary Figure 2), which are known to be H_2_O_2_-responsive marker genes (Wakao et al., 2014), as well as the increase of activity of superoxide dismutases (SOD) and catalases (CAT), which are the dominant ROS scavenging enzymes known to be present in the chloroplasts, was less pronounced in *x32* mutant than in wild-type (Supplementary Figure 3). Interestingly, our experimental data shows that *x32* was more resistant to neutral red (NR) and rose bengal (RB) than wild-type (**Figure 4B**, lower panel). These observations raise the possibility that anti-singlet oxygen response is enhanced in *x32* mutant under such stress conditions.

### Up-Regulated *GSTS* Expression in *x32* Mutant

**FIGURE 5:**
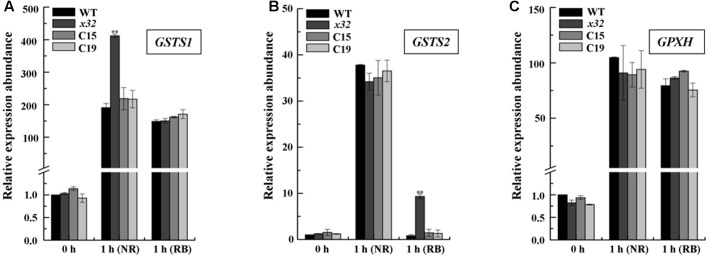
Up-regulated *GSTS* expression in *x32* mutant under singlet oxygen stress. qRT-PCR analysis of expression of *GSTS1* **(A)**, *GSTS2* **(B)**, and *GPXH* **(C)** in WT, *x32* and the complemented strains (C15 and C19) treated with NR and RB, respectively. Standard deviations were estimated from three biological replicates. Similar results were obtained in at least three independent experiments. *CBLP* gene was used as a control. ^∗^*p*-values < 0.05, ^∗∗^*p* < 0.01 in Student’s *t*-test, respectively.

To test the possibility mentioned above, we measured the mRNA levels of several key genes encoding glutathione-*S*-transferases and thioredoxin peroxidase, i.e., *GSTS* and *GPXH*, that are known to be involved in singlet oxygen-induced acclimation process in Chlamydomonas (Leisinger et al., 2001; Fischer et al., 2005, 2009; Ledford et al., 2007) using qRT-PCR. Total mRNA isolated from different strains treated with NR or RB was subjected for the analysis. **Figure 5** shows that expression of these genes was in most cases increased in all strains. The fold-increase of expression in wild-type was similar to the earlier report (Fischer et al., 2005). In *x32*, a differential increase pattern of expression was observed compared to that in wild-type (**Figures 5A–C**). Higher expression of *GSTS1* and *GSTS2* in *x32* was most remarkable under NR (2.3-fold) and RB (8.9-fold) treatment, respectively. Based on these experimental results, we conclude that the singlet-oxygen resistance observed in the mutant could be largely attributed to the up-regulated expression of *GST* genes.

## Discussion

In this work, we have isolated and characterized *x32* mutant lacking CGLD1, a predicted protein belongs to the GreenCut2 superfamily, in Chlamydomonas. We have provided direct experimental evidence of CGLD1 in maintaining structure and function of PSII as well as in protecting Chlamydomonas against photo-oxidative stress. New insights revealed in this work are discussed below.

In the genome of Chlamydomonas. CGLD1 is annotated as a predicted protein with unknown function (Phytozome v12.1^6^). Using a forward genetics approach, we have determined its important and specific role in PSII functionality of this organism (**Figures 1**, **3**). This finding is somewhat different from that reported in Arabidopsis and Synechocystis, showing that not only PSII supercomplex but also PSI complex was significantly reduced (Schneider et al., 2016; Gandini et al., 2017). Indeed, functional and structural analysis of the photosystems, i.e., low temperature fluorescence (77K) emission spectra and light-induced redox kinetics of P700, BN-PAGE and immunoblot quantifications (**Figures 1**, **3**), allows us to propose a crucial role of CGLD1 for maintaining PSII in Chlamydomonas. Also, based on the genomic information, we have generated a specific antibody against CGLD1 and verified the expression of *CGLD1* in wild-type Chlamydomonas (**Figure 2**). These are novel and direct experimental evidence of presence and major function of CGLD1 protein in Chlamydomonas.

Similar to the observations in Arabidopsis and the original *cgld1* mutant (Schneider et al., 2016), which was derived from a different wild-type Chlamydomonas strain (Dent et al., 2015), supplementation of excess Mn^2+^ rather than Ca^2+^ could largely restored the photosynthetic activity (*F*_v_/*F*_m_) in *x32* mutant (Supplementary Figure 1). These consistent results obtained from different Chlamydomonas strains as well as the cyanobacterium Synechocystis (Brandenburg et al., 2017; Gandini et al., 2017), strongly support the suggestion that CGLD1 is involved in uptake/maintenance of Mn^2+^ homeostasis in photosynthetic organisms. Nevertheless, how this is exactly elicited in chloroplast and cyanobacteria remains an open question. Although direct experimental evidences are currently lacking it is presumed that in Arabidopsis the homolog of CGLD1 (PAM71) functions in Mn^2+^ uptake into thylakoids for optimal PSII performance (Schneider et al., 2016). In Chlamydomonas, it is not fully understood how deletion of CGLD1 impaires PSII under normal growth condition. Based on the similar photosynthetic phenotype of Chlamydomonas and Arabidopsis in response to excess Mn^2+^ (Supplementary Figure 1; Schneider et al., 2016), it could be postulated that CGLD1 protein in Chlamydomonas functions in the similar way that proposed for its homolog in Arabidopsis (Schneider et al., 2016) under normal growth condition.

No information is available so far characterizing the expression of CGLD1 protein under adverse conditions including high-light irradiation and oxidative stress. Our finding of elevated level of CGLD1 protein in wild-type Chlamydomonas cells after these treatments (**Figure 4**) implicates its putative function in photo-oxidative responses. Physiological significance of CGLD1 under these stress conditions was demonstrated by the distinct phenotype of wild-type and *x32* (**Figure 4**). Based on the increased sensitivity of the mutant to high-light and peroxide stress, we propose that CGLD1 is also involved in photoinhibition or photodamage/repair of PSII. Since D1 protein is widely accepted as the primary target damaged during photoinhibition *in vivo* (Mulo et al., 2012) and a repair mechanism which involves an intricate and multi-step process operates in all photosynthetic organisms (Nixon et al., 2010; Nickelsen and Rengstl, 2013), we could speculate that deletion of CGLD1 may impact efficient PSII repair (including D1 synthesis) in the organism. Further research is directed toward in-depth understanding of the underlying molecular mechanisms.

Interestingly, we found different response patterns of *x32* to photo-oxidative stress in comparison with wild-type Chlamydomonas. While higher sensitivity of the mutant to high-light and peroxide stress, as frequently reported for PSII mutants as well as the reduced SOD and CAT activity in *x32* treated with H_2_O_2_ (Supplementary Figure 3), was expected, the increased tolerance to singlet-oxygen stress was for the first time revealed in a Chlamydomonas mutant lacking CGLD1 (**Figure 4**). The reason for this novel phenotype of *x32* is currently unclear. In *sor1* mutant, which lacks a basic leucine zipper transcription factor, the singlet-oxygen resistance has been mainly attributed to up-regulated expression of the stress responsive genes *GSTS* and *GPXH* in Chlamydomonas (Fischer et al., 2012). Our finding of up-regulated *GSTS* expression in *x32* under singlet oxygen stress (**Figure 5**) is of indication that the detoxifying system described in *sor1* is also reinforced in this mutant under such stress conditions. Moreover, it has been previously reported that non-photochemical quenching (NPQ) was increased compared to the wild-type (Dent et al., 2015). Considering that NPQ is one of the efficient modules in photoprotection, we would presume that the NPQ mechanism is also enhanced in *x32* mutant lacking CGLD1 and contributes at least partially to its increased resistance to singlet oxygen stress.

In summary, the present work determined expression and functional significance of CGLD1 in Chlamydomonas. Loss of CGLD1 leads to minimal photoautotrophic growth and PSII activity in *x32* mutant. Biochemical analysis of *x32* revealed that the steady levels of PSII supercomplex and core proteins was dramatically reduced compared to wild-type. Furthermore, we found that *x32* was, due to loss of CGLD1, more tolerant to singlet oxygen stress than wild-type. Correlated with this, up-regulated *GSTS* expression was found increased more in *x32*. The phenotypical and physiological implications revealed from this study provide important information for in-depth studies toward understanding structure and function of CGLD1 in Chlamydomonas as well as a valuable alga strain with increased resistance to singlet oxygen stress for potential applications.

## Author Contributions

JX and FH conceived the research and designed the experiments. JX, PL, and LZ performed the experiments. JX and FH analyzed data and wrote the manuscript.

## Conflict of Interest Statement

The authors declare that the research was conducted in the absence of any commercial or financial relationships that could be construed as a potential conflict of interest.

## Footnotes

1 http://www.chlamycollection.org

2 http://frodo.wi.mit.edu/

3 https://giavap-genomes.ibpc.fr/cgi-bin/predalgodb.perl?page=main

4 http://www.chlamycollection.org

5 https://phytozome.jgi.doe.gov

6 https://phytozome.jgi.doe.gov
