# Supplementary material for: Deletion of CGLD1 Impairs PSII and Increases Singlet Oxygen Tolerance of Green Alga Chlamydomonas reinhardtii
Source: Front Plant Sci. 2017 Dec 15;8:2154. doi: 10.3389/fpls.2017.02154 (PMC5736878; doi:10.3389/fpls.2017.02154)
Supplement: Supplementary file 1 [file Table_1.DOC]

Supplemental Table 1. List of primers used in this study

| Primer | Nucleotide sequences (5’ to 3’ ) | Purpose |
| --- | --- | --- |
| Sp0 | AGGACCCGTGGTTCGGGCCGGAGTGTTC | Tail-PCR |
| Sp1 | GCGATGGACTCCAGTCCGGCCTTCCGCGGTGTTCCTGTGGGAGTAC |
| LAD1 | ACGATGGACTCCAGAGCGGCCGCVNVNNNGGAA |
| LAD2 | ACGATGGACTCCAGAGCGGCCGCBNBNNNGGTT |
| LAD3 | ACGATGGACTCCAGAGCGGCCGCVVNVNNNCCAA |
| LAD4 | ACGATGGACTCCAGAGCGGCCGCBDNBNNNCGGT |
| LAD5 | ACGATGGACTCCAGAGCGGCCGCNTCGWGWTSCNAGC |
| AC1 | ACGATGGACTCCAGAG |
| AF, AR | CTAGGTCTTTGGCCTCAGA, TTGGCTGTGGAGAAGAAGAT | Mapping of *aphVIII* insertion site in genome of *x32* |
| BF, BR | TGCTCCCGTGTACGATGAG, ACCACGCGGTTTAAGCGAC |
| CF, CR | TCGTGGGCGACTCGCCGCT, AATCAGTTGGGACAGCACC |
| DF, DR | AGATGGATCCTGGGGAGGG, CATTGCCCACGACTGTGGA |
| EF, ER | GTATGCCTAGTATTGAAAC, CTGCGGGTGGATCTGTGCGC |
| FF, FR | GCTGGCTGGCGCCGGCTGGA, GCTGTACCTCCCACCACA |
| GF, GR | GCTATGGCATGTTCATTCG, AGTCGTAGTGGGCTCCGTA |
| *CGLD1*-COM-F/R | GAATTCTTACACGAACAGGTCCACGATGG, CATATGAGCTGTGCAAACCTACTCAAAAG | Complementation constructs |
| Mt+Fus1-F/R | ATGCCTATCTTTCTCATT, GCAAAATACACGTCTGGAAG | Mating-type determination |
| Mt-Mid-F/R | ATGGCCTGTTTCTTAGC, CTACATGTGTTTCTTGAC |
| *AphVIII*-F/R | CTTGTTGGGTGAGGCTGAGC, GGCACAGGTGACCGTGGCAAAC | DNA blot probe amplification |
| Pet28-*CGLD1*-F/R | CATATGCAGGTGGACGAGCTGGTGCC,  GAATTCGCCCAGCGGCGAGGAGGCCG | Recombinant CGLD1 expression |
| *psbA*-F/R | GTATTTGGTTCACTGCTTTAG, GGAAGTTGTGAGCGTTACGC | qRT-PCR |
| *psbC*-F/R | TTCCCGTACTTTGTATCAGG, TGCACCTAAGCCTAACATGA |
| *GPXH*-F/R | ACGTGTTTGACACGGTTATGAGA, GCAATTGGCATGATGGATAGTG |
| *GSTS1*-F/R | CAGAGGTGAAAGGCGGATAC, GTGTTGCAATGGACTTCAGC |
| *GSTS2*-F/R | ACCCCATCGGCAAAACAAG, CCGGTATAGTATTGTAGCAGAATAACCA |
| *CBLP*-F/R | ATGACCACCAACCCCATCATC, GGTCCCACAGCATGGCAATG |
| *APX1*-F/R | TCAAGGAGATCAAGGCCAAG,  GCCGCTCAGTCCAGAGTAAC |
| *CAT1*-F/R | CACGAGGGCTTCATGAACTT,  CTTGGCGATCACCATCTTCT |
