# Supplementary material for: Deletion of CGLD1 Impairs PSII and Increases Singlet Oxygen Tolerance of Green Alga Chlamydomonas reinhardtii
Source: Front Plant Sci. 2017 Dec 15;8:2154. doi: 10.3389/fpls.2017.02154 (PMC5736878; doi:10.3389/fpls.2017.02154)
Supplement: Supplementary file 2 [file Image_1.PDF]

Supplemental Figure 1

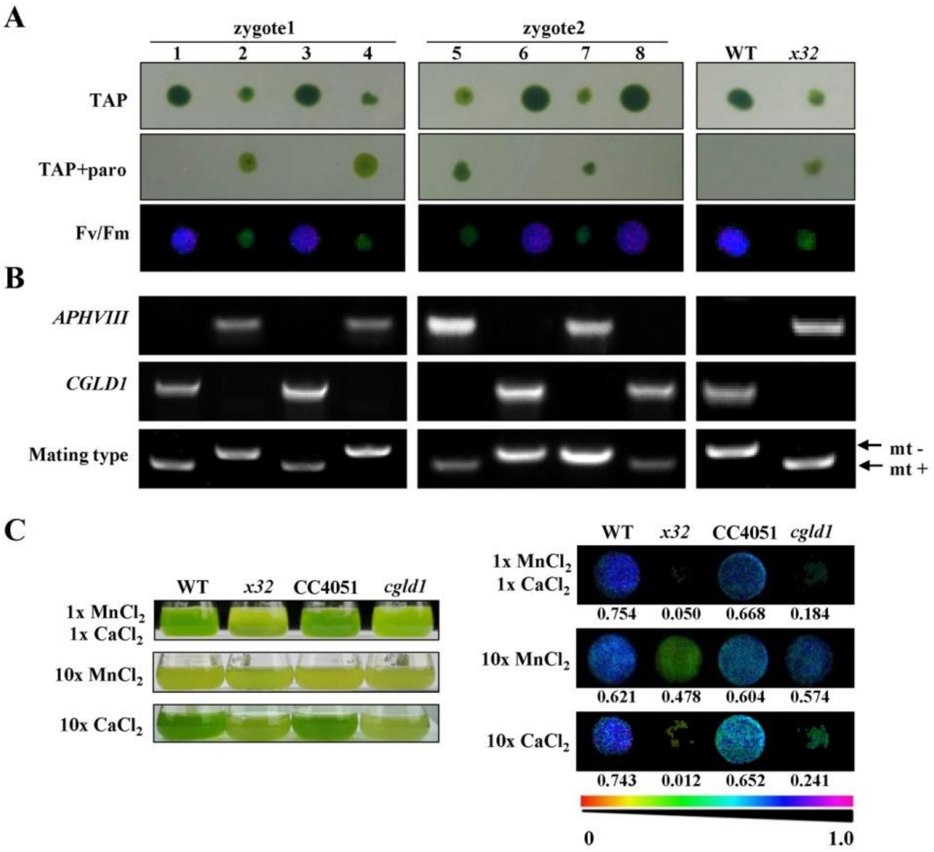

Supplemental Figure 1. Tetrad and phenotypic analysis of CGLD1-deficient mutants. **(A)** Tetrad analysis of a cross between *x32* and wild type (CC400). Growth patterns of the representative tetrads on TAP and TAP containing paromomycin are shown. Fv/Fm values of the representative tetrads on TAP are shown with a false color scale. **(B)** PCR analysis of the representative tetrads shown in **(A)** using *AphVIII*-F/R primers for detection of the paromomycin resistance cassette (*APHVIII*), using CF/CR primers for detection of *CGLD1* gene, using the primers (Mt+Fus1-F/R, Mt-Mid -F/R) for determination of mating-type, respectively. **(C)** Comparision of growth and Fv/Fm of the indicated strains in the presence of excess Mn^{2+} and Ca^{2+} concentrations. Cells were grown in TAP medium with normal (1x $MnCl_2$, $CaCl_2$) and excess (10x $MnCl_2$ or $CaCl_2$) concentrations for 3 days, respectively. Fv/Fm values of the cells are shown with a false color scale.
