# Supplementary material for: Deletion of CGLD1 Impairs PSII and Increases Singlet Oxygen Tolerance of Green Alga Chlamydomonas reinhardtii
Source: Front Plant Sci. 2017 Dec 15;8:2154. doi: 10.3389/fpls.2017.02154 (PMC5736878; doi:10.3389/fpls.2017.02154)
Supplement: Supplementary file 3 [file Image_2.PDF]

Supplemental Figure 2

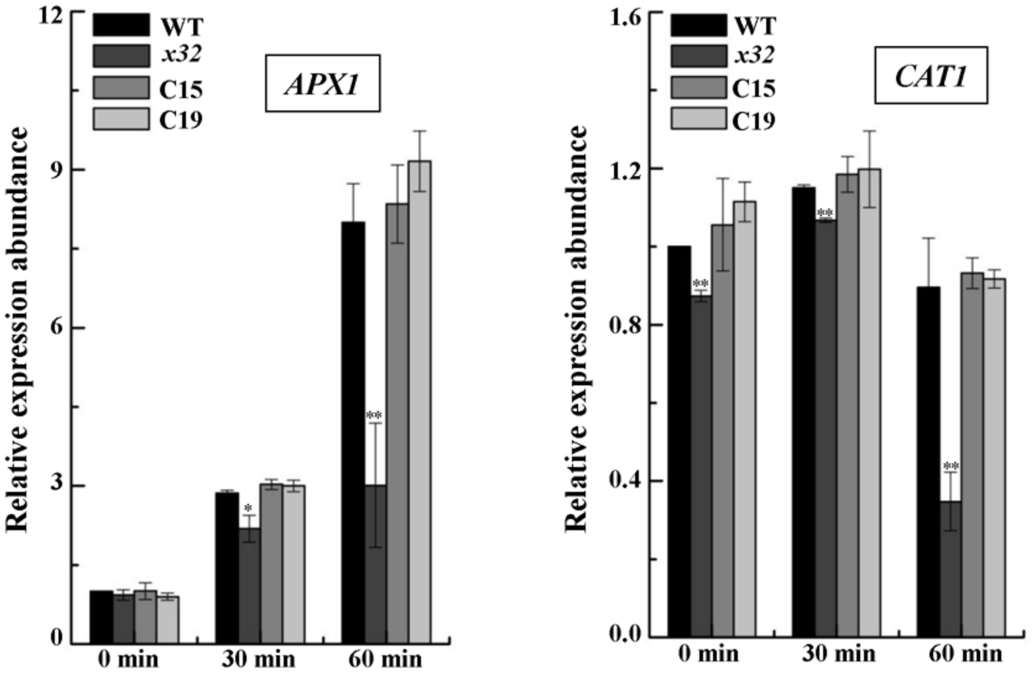

Supplemental Figure 2. Down-regulated H_2O_2-**responsive gene expression in *x32* mutant after treatment with H_2O_2.** qRT-PCR analysis of expression of *APX1* and *CAT1* in the indicated strains. Standard deviations were estimated from three biological replicates. Similar results were obtained in three independent experiments. *CBLP* gene was used as a control. * and ** refer to p-values <0.05 and <0.01 in Student's t-test, respectively.
