# Supplementary material for: Deletion of CGLD1 Impairs PSII and Increases Singlet Oxygen Tolerance of Green Alga Chlamydomonas reinhardtii
Source: Front Plant Sci. 2017 Dec 15;8:2154. doi: 10.3389/fpls.2017.02154 (PMC5736878; doi:10.3389/fpls.2017.02154)
Supplement: Supplementary file 4 [file Image_3.PDF]

Supplemental Figure 3

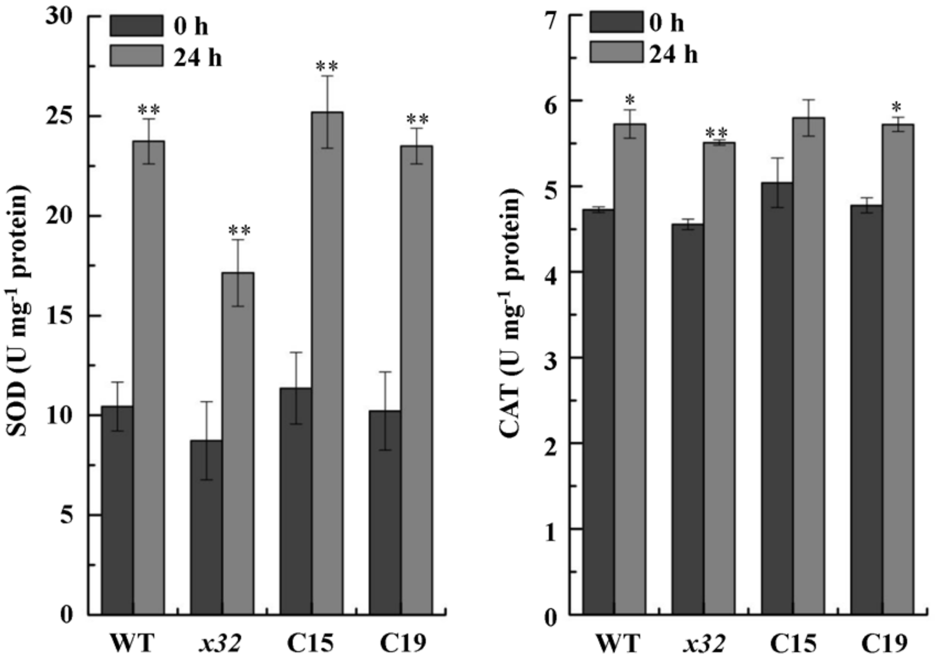

Supplemental Figure 3. Activity of SOD and CAT in the indicated strains after treatment with H_2O_2. Data are presented as means ± SD. Standard deviations were estimated from three biological replicates. All experiments were repeated more than three times with similar results. * and ** refer to p-values <0.05 and <0.01 in Student's t-test, respectively.
